# Dementia with Lewy bodies: an update and outlook

**DOI:** 10.1186/s13024-019-0306-8

**Published:** 2019-01-21

**Authors:** Tiago Fleming Outeiro, David J. Koss, Daniel Erskine, Lauren Walker, Marzena Kurzawa-Akanbi, David Burn, Paul Donaghy, Christopher Morris, John-Paul Taylor, Alan Thomas, Johannes Attems, Ian McKeith

**Affiliations:** 1 Institute of Neuroscience, The Medical School, Newcastle University, Framlington Place, Newcastle Upon Tyne, NE2 4HH UK; 2 Department of Experimental Neurodegeneration, Center for Nanoscale Microscopy and Molecular Physiology of the Brain, Center for Biostructural Imaging of Neurodegeneration, University Medical Center Göttingen, Göttingen, Germany; 3 Max Planck Institute for Experimental Medicine, Göttingen, Germany

**Keywords:** Dementia with Lewy bodies, Alpha-synuclein, Dementia, Alzheimer’s disease, Biomarkers

## Abstract

Dementia with Lewy bodies (DLB) is an age-associated neurodegenerative disorder producing progressive cognitive decline that interferes with normal life and daily activities. Neuropathologically, DLB is characterised by the accumulation of aggregated α-synuclein protein in Lewy bodies and Lewy neurites, similar to Parkinson’s disease (PD). Extrapyramidal motor features characteristic of PD, are common in DLB patients, but are not essential for the clinical diagnosis of DLB. Since many PD patients develop dementia as disease progresses, there has been controversy about the separation of DLB from PD dementia (PDD) and consensus reports have put forward guidelines to assist clinicians in the identification and management of both syndromes. Here, we present basic concepts and definitions, based on our current understanding, that should guide the community to address open questions that will, hopefully, lead us towards improved diagnosis and novel therapeutic strategies for DLB and other synucleinopathies.

## Synucleinopathies: a general overview

The synucleinopathies comprise several neurodegenerative disorders characterised by the accumulation of aggregated forms of the protein α-synuclein (α-syn) in both neuronal and non-neuronal cells in the brain. Most idiopathic synucleinopathies are age-associated and, therefore, their prevalence is increasing in parallel with the world wide increase in life expectancy [1]. Synucleinopathies are second to Alzheimer’s disease (AD) amongst the most common neurodegenerative disorders known to cause dementia [2]. As with most neurodegenerative disorders, there are still no disease-modifying drugs, limiting treatment options to symptomatic relief and palliative measures. Therefore, synucleinopathies pose a growing socio-economic burden to modern societies, and demand urgent attention.

Most synucleinopathies are Lewy body diseases (LBD), as they are characterised by the accumulation of aggregated a α-syn into Lewy bodies (LBs) within vulnerable neurons and Lewy neurites (LN) in neuronal processes [3]. The LBD comprise Parkinson’s disease (PD), Parkinson’s disease dementia (PDD), and dementia with Lewy bodies (DLB), among other less common disorders [4]. The central role of α-syn in LBD originated from almost simultaneous findings of mutations in the gene encoding for α-syn (*SNCA*) in familial forms of PD [5], and of α-syn comprising the major protein component of Lewy bodies [3].

Multiple system atrophy (MSA) is neuropathologically characterised by accumulation of aggregated α-syn in oligodendrocytes, inclusions known as glial cytoplasmic inclusions (GCIs) [4, 6], while LB pathology is absent and, therefore, MSA is not an LBD.

The initial clinical and neuropathological studies which established the distinct clinical and neuropathological phenotype of the disorder now known as DLB, preceded immunohistochemical methods to detect α-syn in human brain tissue, but later revisions of international consensus for diagnostic guidelines now recommend the use of immunohistochemistry [7–11].

Clinical under-diagnosis of DLB [12], and over-diagnosis of PD [13, 14], have led to most studies of LBD focusing on PD and PDD, leaving DLB historically under-researched relative to its population prevalence. Increasing recognition of DLB as a distinct and prevalent age-associated neurodegenerative dementia has stimulated increasing numbers of high-quality studies on its aetiology and pathogenesis. Here, we summarise contemporary findings from this rapidly expanding field, focusing on genetics, diagnostic biomarkers and molecular mechanisms.

## The clinical definition of DLB

DLB is now the preferred term [8, 10, 11] for a variety of previously used clinical diagnoses including diffuse LB disease (DLBD) [15–17], LB dementia [18], dementia associated with cortical Lewy bodies (DCLB) [19], the LB variant of Alzheimer’s disease (LBVAD) [20, 21], and senile dementia of LB type (SDLT) [22].

Recognition and definition of the DLB syndrome originally occurred through *post-mortem* neuropathological observations, of a particular distribution of LB and LN in the brains of elderly subjects with dementia, followed by a retrospective review of their clinical histories [23]. This revealed two major findings – the first was that a significant number of LB pathology cases had a clinical presentation that was discernibly different from other dementia subtypes, even at an early stage in the disease. Fluctuating levels of cognitive impairment, recurrent visual hallucinations, spontaneous extrapyramidal motor features and a history of rapid eye movement (REM) sleep behavior disorder (RBD) were the most prominent symptoms, and the presence of two or more of these symptoms in an individual with dementia is now considered sufficient for a clinical diagnosis of probable DLB.

The other major observation was that approximately 50% of subjects showing full blown DLB pathology at neuropathological *post-mortem* examination did not show the characteristic clinical picture of DLB during life but typically presented with global cognitive decline reminiscent of AD. Unsurprisingly, such cases usually show additional high levels of AD neuropathological change [24, 25]. The true prevalence of such mixed pathology cases is unknown but autopsy studies indicate that between a third and a half of carefully clinically diagnosed AD show at least some degree of LB pathology at autopsy [20, 26]. Complex visual hallucinations are the only clinical feature indicating the likely presence of LB pathology in an otherwise typical AD case [27], but robust data on progression, prognosis and response to treatments of “mixed AD+DLB” (*i.e.,* cases showing both full blown AD and DLB pathology) are lacking.

While a recent UK estimate found that only 4.6% of specialist dementia service referrals were clinically diagnosed with DLB [28], substantial LB pathology was present in about 20% of *post-mortem* brains, further underpinning the general under-diagnosis of DLB during life. Moreover, there was substantial variability in DLB clinical diagnosis rates (2.4% - 5.9%) between individual clinicians working in geographically proximal services suggesting that performance could be improved simply by better application of clinical methods and by increased use of biomarkers (see section "Biomarkers in LBD").

The current clinical diagnostic criteria for DLB are shown in Table 1. Dementia, defined as a progressive cognitive decline of sufficient magnitude to interfere with normal social or occupational functions, or with usual daily activities, is an essential requirement. Disproportionate attentional, executive function and visual processing deficits relative to memory and naming are typical features. Diagnostic toolkits have been published to assist clinicians to identify the core clinical features [29–31] but no DLB-specific cognitive batteries have yet been developed.

**Table 1:** Revised criteria for the clinical diagnosis of probable and possible DLB

**Essential** for a diagnosis of DLB is dementia, defined as a progressive cognitive decline of sufficient magnitude to interfere with normal social or occupational functions, or with usual daily activities. Prominent or persistent memory impairment may not necessarily occur in the early stages but is usually evident with progression. Deficits on tests of attention, executive function and visuo-perceptual ability may be especially prominent and occur early. **Core clinical features** *(the first three typically occur early and may persist throughout the course)* Fluctuating cognition with pronounced variations in attention and alertness. Recurrent visual hallucinations that are typically well formed and detailed. REM sleep behaviour disorder (RBD) *which may precede cognitive decline*. One or more spontaneous cardinal feature of parkinsonism – these are bradykinesia (defined as slowness of movement and decrement in amplitude or speed), rest tremor, or rigidity. **Supportive clinical features** Severe sensitivity to antipsychotic agents ; postural instability ; repeated falls ; syncope or other transient episodes of unresponsiveness.; severe autonomic dysfunction e.g. constipation, orthostatic hypotension, urinary incontinence ; hypersomnia; hyposmia; hallucinations in other modalities; systematized delusions; apathy, anxiety and depression. **Indicative biomarkers** Reduced dopamine transporter (DaT) uptake in basal ganglia demonstrated by SPECT or PET Abnormal (low uptake) MIBG myocardial scintigraphy Polysomnographic confirmation of REM sleep without atonia **Supportive biomarkers** Relative preservation of medial temporal lobe structures on CT/MRI scan Generalised low uptake on SPECT/PET perfusion/metabolism scan with reduced occipital activity +/- the cingulate island sign on FDG-PET imaging Prominent posterior slow wave activity on EEG with periodic fluctuations in the pre-alpha/theta range ***Probable DLB*** *can be diagnosed if:* * a) two or more core clinical features of DLB are present, with or without the presence of indicative biomarkers or* * b) only one core clinical feature is present, but with one or more indicative biomarkers* ***Probable DLB*** *should not be diagnosed on the basis of biomarkers alone* ***Possible DLB*** *can be diagnosed if:* * a) only one core clinical feature of DLB is present, with no indicative biomarker evidence, or* * b) one or more indicative biomarkers is present but there are no core clinical features* **DLB** is less likely**:** a) in the presence of any other physical illness or brain disorder including cerebrovascular disease, sufficient to account in part or in total for the clinical picture, although these do not exclude a DLB diagnosis and may serve to indicate mixed or multiple pathologies contributing to the clinical presentation. *or* b) if parkinsonian features are the only core clinical feature and appear for the first time at a stage of severe dementia. DLB should be diagnosed when dementia occurs before, or concurrently with parkinsonism. The term Parkinson’s disease dementia (PDD) should be used to describe dementia that occurs in the context of well-established Parkinson’s disease. In a practice setting the term that is most appropriate to the clinical situation should be used and generic terms such as LB disease are often helpful. In research studies in which distinction needs to be made between DLB and PDD the existing one-year rule between the onset of dementia and parkinsonism continues to be recommended.	

Adapted from [11]

The item generally causing the most difficulty in assessment is the identification of cognitive fluctuation. It is recommended to use one of several published methods which typically use a series of structured questions asking: (i) about changes in the patient’s level of functioning during the day; (ii) about excessive daytime drowsiness; or (iii) about difficulty in arousing the patient so they maintain attention throughout the day. RBD can be difficult to differentiate from the numerous other sleep disturbances that can occur in dementia unless the care-taker is specifically asked whether they have ever seen the patient appear to "act out his/her dreams" while sleeping (punching or flailing arms in the air, shouting or screaming). Assessment of parkinsonism can be problematic, especially when the clinician is not an expert movement disorder neurologist, since motor features may be absent in up to 25% of autopsy confirmed DLB cases and, even when present, may be very mild. Documentation of only one of the cardinal features, bradykinesia, resting tremor, or rigidity, is required for DLB, while at least two are required to diagnose PD. Co-morbidities, e.g. arthritis, or inability to comply with neurological examination because of cognitive impairment may lead to false positive diagnoses.

Recurrent, complex visual hallucinations, which occur in the majority of DLB patients, pose less problems of recognition, provided that the clinician asks directly about them and quantifies their severity with an appropriate scale. They are typically well formed, featuring people or animals, and may be accompanied by related phenomena including passage hallucinations, sense of presence and visual illusions. Patients are typically able to report these experiences, as are observant caregivers [23].

A case of probable DLB established using consensus criteria has been estimated as having a diagnostic specificity at autopsy of ~85%, possibly the highest of the common neurodegenerative dementia subtypes. The extent to which the addition of indicative biomarkers in the revised DLB criteria will increase this specificity, remains to be determined [32].

Additional clinical features are known to be supportive of a DLB diagnosis. These are symptoms that are commonly present, sometimes early [33] and which may indicate DLB in a patient with dementia, particularly when they persist over time or if several occur in combination (Table 1).

Another important issue to consider is the relationship between the diagnosis of DLB and that of dementia occurring in a patient with a pre-existing clinical diagnosis of PD, usually referred to as PDD. This has been a source of controversy and, therefore, needs clarification and continued research efforts. Although the end stage neuropathological findings in such cases may be similar, there can be little doubt that the clinical experience of the patients and their families will have been very different. DLB is typically a disorder associated with cognitive impairment in which extrapyramidal motor features are often mild or absent, at least until the late stages. In contrast, PDD is characterised by early and prominent extrapyramidal motor features required for PD diagnosis, with neuropsychiatric and cognitive symptoms occurring later. Undoubtedly, the two distinct clinical syndromes of DLB and PD/PDD share underlying pathomechanisms and, while the reasons for the clinical heterogeneity may be due to different propagation patterns of α-syn pathology across different neuronal pathways, the additive effects of concomitant AD pathology which is more common and severe in DLB as compared to PD/PDD should be taken into consideration. Hence, it is inappropriate to simply use PD as an umbrella term for all LBD, and this is reflected in the original formulation of the “one-year rule” (bottom of Table 1) by which DLB should be diagnosed when dementia occurs before, or concurrently with parkinsonism, while the term PDD should be used to describe dementia that occurs in the context of well-established PD ([34] for further discussion). This approach, adopted by DSM5 [35] and the final draft of ICD-11 [36], both of which recommend the distinction of DLB and PDD, suggests that this convention will remain in use until new scientific insight allows to distinguish between DLB and PD/PDD based on specific and well characterized differences in their respective pathomechanisms.

The mean age of onset of PDD and DLB is similar at >70 years whereas PD onset is typically earlier with a mean of 60 years. Data regarding the comparative age related prevalence of PDD and DLB are limited with some suggesting that DLB patients are younger at symptom onset than those with PDD and with more hallucinations and cognitive fluctuations; and others reporting younger age at disease onset in PDD or no essential differences between disorders [37].

## Biomarkers in DLB

The diagnostic criteria of DLB identify ‘indicative’ and ‘supportive’ biomarkers based upon their diagnostic specificity and the volume of good quality evidence available (Table 1) [11]. The presence of an indicative biomarker in combination with a single core clinical feature is sufficient for a diagnosis of probable DLB. Supportive biomarkers are consistent with DLB but lack the specificity of the indicative biomarkers.

### Indicative biomarkers

#### Striatal dopamine transporter imaging

Like PD, DLB is associated with nigrostriatal dopaminergic neuron loss. This can be detected using SPECT or PET imaging using a ligand that binds to presynaptic dopamine transporters (e.g. N-ω-fluoropropyl-2β-carbomethoxy-3β-(4-iodophenyl) nortropane (FP-CIT)). Visually rated FP-CIT SPECT has a sensitivity of 78% and specificity of 90% to differentiate probable DLB from other dementias when compared with clinical diagnosis [38]. This has been confirmed with post-mortem diagnosis [39]. The upper limit of sensitivity of FP-CIT SPECT reflects the absence of substantia nigra pathology sufficient to cause an abnormal scan in some cases of DLB [40, 41].

**Fig. 1:**
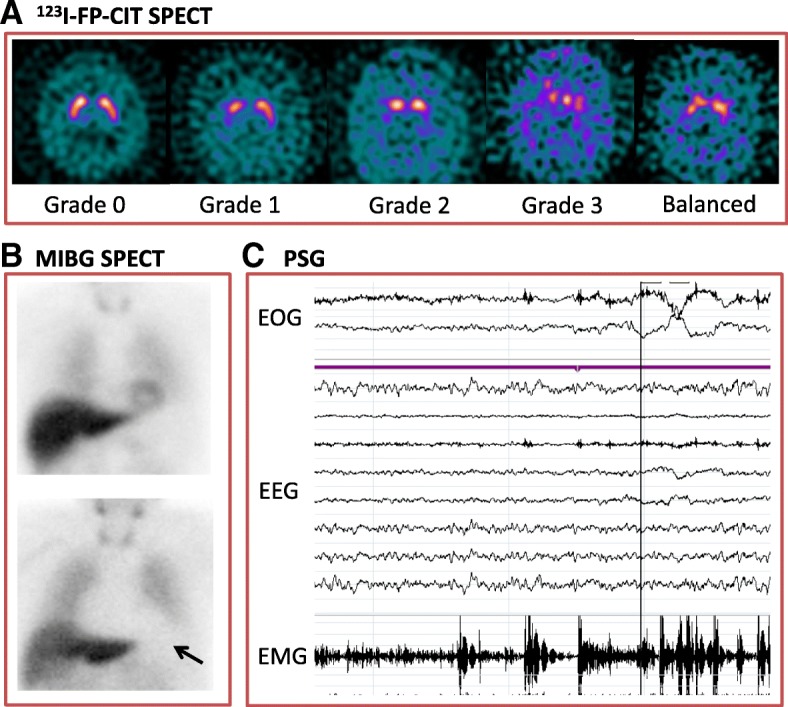
Indicative biomarkers for dementia with Lewy bodies. A. N-ωfluoropropyl-2β-carbomethoxy- 3β-(4-iodophenyl) nortropane (123I-FP-CIT SPECT) single photon emission tomography (SPECT). Axial images from FP-CIT SPECT at the level of the striatum. Grade 0 – normal uptake in left and right striatum. Grade 1 – unilateral decreased uptake in putamen [42]. Grade 2: bilateral uptake in putamen. Grade 3: virtually absent uptake bilaterally in the caudate and putamen. Balanced bilateral loss in the caudate and putamen is often seen in DLB, which does not fit easily into any Benamer scale category. B. Cardiac Meta-iodobenzylguanidine (MIBG SPECT) Imaging. The top image is normal, with a clear cardiac outline visible (arrow, HMR=3.14). The bottom image is abnormal with no visible cardiac outline (HMR=1.03). C. Polysomnography (PSG) recording demonstrating episodes of REM sleep without atonia on electro-oculogram (EOG) measuring eye movements, electroencephalogram (EEG) and electromyogram (EMG) measuring chin movement. With thanks to Dr Sean Colloby (**a**), Ms Gemma Roberts (**b**) and Dr Kirstie Anderson (**c**)

FP-CIT SPECT images can be rated visually using a scale developed for PD [42], though many cases of DLB are difficult to classify using this scale (Fig. 1a) [43]. Clinical reports often use a combination of visual interpretation and semi-quantitative analysis, which has been shown to increase reader confidence [44, 45].

Dopamine transporter imaging should not be used to differentiate DLB from frontotemporal dementia, progressive supranuclear palsy, corticobasal syndrome or multiple system atrophy as these conditions can also be associated with reduced striatal dopamine transporters [46].

#### MIBG Myocardial scintigraphy

Cardiac autonomic denervation is found in Lewy body diseases such as PD, DLB and pure autonomic failure [47]. Meta-iodobenzylguanidine (MIBG) is a noradrenaline analogue that binds to presynaptic cardiac autonomic nerve terminals. MIBG binding in the heart is compared to non-specific binding in the mediastinum (H:M ratio, Fig. 1b). Single centre studies have demonstrated high sensitivity and specificity of MIBG scintigraphy [48–50]. The only multicentre study to date found a sensitivity of 69% and specificity of 89% [51]. The diagnostic accuracy of MIBG in this study improved when compared with clinical diagnosis 3 years after the scan (sensitivity 77%, specificity 97%) [52].

A limitation of MIBG scintigraphy is that comorbid conditions and medications can reduce cardiac MIBG uptake [53]. As a result, studies have excluded participants with common conditions such as heart failure, ischaemic heart disease and poorly-controlled diabetes [51]. Thresholds for abnormality in H:M ratio differ between centres, due in part to differences in collimators (from 1.60 to 2.20 in the above studies) [48, 52]. Individual centres should therefore develop local thresholds prior to clinical application.

#### Polysomnography

Polysomnography (Fig. 1c) allows for the objective identification of RBD by measuring EEG, eye movements and muscle movement during sleep. Polysomnography-confirmed RBD has a very high diagnostic specificity of 98% for synucleinopathies (PD, DLB or MSA) [54]. It had an 84% sensitivity in post-mortem confirmed DLB cases [55]. Sensitivity may be limited by the absence of REM sleep in some polysomnography sessions.

### Supportive biomarkers

#### Imaging

DLB is associated with less severe medial temporal lobe atrophy on structural imaging when compared to AD [56]. However, the sensitivity of this biomarker to detect DLB is limited by the presence of AD pathology and associated medial temporal lobe atrophy in a significant proportion of DLB cases [57]. Occipital hypoperfusion and hypometabolism can also be seen on functional imaging, though FDG-PET is more effective in identifying DLB than HMPAO-SPECT [58].

#### EEG

DLB is characterised by the presence of prominent posterior slow wave activity [59–61] and temporal slow wave activity [62]. The dominant EEG rhythm, normally within the alpha range, is slowed toward pre-alpha/fast theta and the variability of dominant frequency over time is increased [60, 61, 63, 64]. Single centre studies have reported good to excellent discrimination of DLB from AD using quantification of EEG by a variety of methods [59]; multicentre studies have been more equivocal [60]. However, EEG may be an important biomarker for DLB in the future as changes can be detected early in the disease course [65, 66].

### Other biomarkers

#### Fluid biomarkers

CSF α-syn levels have variously been found to be increased, decreased or unchanged in DLB [67]. The reasons for these conflicting results may include contamination (e.g. with blood) and differences in CSF acquisition, processing and analysis [68]. The differentiation of DLB from AD using CSF markers is further complicated by the presence of AD pathology in a significant proportion of DLB cases as discussed above. At present, CSF measures cannot discriminate between DLB and AD, but markers of AD pathology may be useful in stratifying DLB patients for future clinical trials [69].

#### Novel Biomarkers

The development of biomarkers for the diagnosis of LBD such as DLB is an active area of research. Much of this effort is focussed on the development of an α-syn biomarker to complement the β-amyloid (Aβ) and tau biomarkers that have been developed for AD. α-syn imaging ligands are currently in the pre-clinical stage [70]. α-syn biomarkers in other tissues such as skin [71], and gut [72] are currently being investigated.

### Genetics of DLB

Our present understanding of the genetic aetiology of DLB is limited. Nevertheless, the available studies suggest that genetic factors are as important in DLB as in AD or PD. Positive family history of dementia and DLB is a strong risk factor for DLB and siblings of affected individuals are at 2.3 fold risk of developing the disease themselves [73, 74]. Nonetheless, DLB pedigrees with highly penetrant alleles are rare and frequencies of genetic variants in genes linked with DLB are poorly understood.

Whilst families with DLB are rare, such families are informative in providing genetic insight to the aetiology of DLB. Most cases of suggested familial DLB show a predominant PD phenotype where many family members have motor impairment as a presentation long before onset of cognitive symptoms. Very few families with suggested DLB show cognitive problems at presentation. Consequently many families, while they do show cognitive changes and dementia as part of the disease process, do not have typical DLB meeting consensus criteria in all family members. For example, individuals in families with rare point mutation in the SNCA gene such as the Contursi kindred [75, 76] often have profound dementia as part of the disease process, although this is variable and often a later symptom. Typically, cases with point mutations in SNCA present as early onset PD [77–79]. Similarly, in the Waters-Miller-Muenter kindred with triplication of SNCA [80], onset is typically motor impairment with very few cases showing cognitive impairment at presentation and which can be described as having DLB [81]. Families with SNCA duplication do present clinically with certain features of DLB and show typical pathology of neocortical α-syn deposition, but again, dementia is often a later feature or not prominent [82–87]. Therefore, SNCA mutations are not a common finding in DLB [88].

Often families and individuals that have AD and causal mutations in APP or PSEN1 along with concurrent presence of LBs, typically in the amygdala, have been described as having DLB or LBD. While these cases fit with a wider view of LBD, most do not meet consensus clinical criteria for DLB [89].

There are however families which do meet clinical criteria for DLB and where familial inheritance is shown. In a description of two families with typical late onset dementia showing typical DLB, analysis showed widespread neocortical α-syn pathology with typically only mild AD pathology, although a genetic defect was not identified [90]. One family with dementia at onset and later development of parkinsonism was reported where age at onset of dementia was variable [91]. Neuropathology of the proband showed widespread neocortical type α-syn pathology and Braak stage V neurofibrillary tangles fulfilling neuropathologic criteria for both DLB and AD. Sequence analysis of this family has shown the presence of a P123H SNCB mutation near the C-terminus of the protein, although no deposition of β-synuclein protein in brain tissue was observed [92].

Two unrelated families with suggested DLB have been reported as carrying a mutation in the EIF4G gene [93] known to be associated with increased risk of PD [94]. In these affected families, presentation was typically a dementia syndrome with variable parkinsonian features and pathology indicative of diffuse neocortical α-syn deposition with only age related AD pathology. Siblings with clinically and neuropathologically confirmed DLB have been reported [95, 96]. However, a shared genetic mutation has not yet been identified [97]. Individuals with DLB do show potentially causative mutations in certain autosomal dominant or recessive genes associated with other neurodegenerative disorders and individuals with mutations in PARK2, CHMP2B, PSEN2, SQSTM1, EIF4G1, and GIGYF2 have been identified [97].

Although families with SNCA mutations do not show clinical characteristics of DLB, association with the SNCA locus is also strongly apparent in large studies of sporadic DLB [98, 99]. Association with the SNCA gene is not surprising due to the protein product α-syn being present in LB and believed to be central in the pathophysiology of DLB, PD and PDD. However, there seems to be an interesting correlation, with the 3’ of the SNCA gene being associated with the PD phenotype and the 5’ region linking with DLB. This may impact on the gene expression and distribution of LB pathology in the brain.

Multiple studies dissecting the genetic component of DLB have been published to date (for a comprehensive review see [100, 101]), and the genetic landscape of DLB mirrors that of the clinical and neuropathological overlap between DLB, PD and AD. To date, no high penetrance pathogenic mutations have been identified. However, a number of common (>1% in population) and rare genetic risk variants have been established. Genes reported to be associated with DLB are SNCA, LRRK2, PSEN1, PSEN2, APP, SNCB, MAPT, SCARB2, GBA and APOE (Table 2). The finding of rare variants in AD genes (PSEN1, PSEN2 and APP) in cases of dementia, as previously noted, might be in part due to misdiagnosis, particularly when the neuropathological assessment has not been possible. The co-occurrence of LB pathology in AD is common and may influence the disease phenotype towards DLB [102]. The recent genome wide association study confirmed several of the previously reported associations (APOE, SNCA and GBA) and identified a new probable locus CNTN1 [99], providing an unbiased and the most comprehensive study of DLB genetics to date.

**Table 2:** Summary of genetic variants associated with DLB. Single nucleotide polymorphisms (SNP), allele or haplotype are listed. For SNPs rs numbers are provided and amino acid variant stated for exonic mutations

Gene	SNP/allele or Haplotype	Gene product	References
APOE	rs429358 (C130R) / E4 (allele)	Apolipoprotein 4	[97–99, 105]
SNCA	rs7681440 (intronic) rs356182 (intronic) rs104893875 (E46K) rs104893877 (A53T) Duplication	α-synuclein	[77, 78, 82, 98, 99]
GBA	Multiple mutations	β-glucocerebrosidase	[99, 107]
SCARB2	rs6812193 (intronic)	Lysosomal integral membrane protein-2	[98]
MAPT	H1G (haplotype) H2 (haplotype) rs143624519 (A152T) R221Q^a^	Microtubule-associated protein tau	[206–208]
LRRK2	rs34637584 (G2019S)	Leucine-rich Kinase-2	[209]
SNCB	rs104893937 (P123H) rs104893936 (V70M)	β-Synuclein	[91, 92]
PSEN1	rs63749824 (A79V)	Presenilin 1	[207]
PSEN2	rs140501902 (R71W) rs63750048 (A85V) V191E^a^ rs63750110 (D439A)	Presenilin 2	[97, 207, 210]
GRN	rs63750441 (C105R) Multiple variants	Granulin	[207, 211]
PARK2	rs148990138 (P37L) A46S^a^ rs191486604(G430D) rs34424986 (R275W)	Parkin	[97, 207]
PINK1	P138L^a^ rs139226733 (M318L) S499C^a^	PTEN-induced kinase 1	[207]
APP	rs63750264 (V717I) Duplication	Amyloid precursor protein	[89, 212]
GABRB3	rs1426210 (intronic)	Gamma-aminobutyric acid receptor subunit beta-3	[99]
BCL7C/STX1B	rs897984 (intronic)	B-cell CLL/lymphoma 7 protein family member C / Syntaxin 1B	[99]
TREM2	rs143332484 (R62H)	Triggering receptor expressed on myeloid cells 2	[211]
CHMP2B	rs63750818 (I29V)	Charged multivesicular body protein 2B	[97]
SQSTM1	rs200396166 (A33V) P27L^a^	Sequestosome	[97]
EIF4G1	M1134V^a^	Eukaryotic translation initiation factor 4 gamma 1	[97]
GIGYF2	S1029C^a^ S66T^a^	GRB10 interacting GYF protein 2	[97]

^a^= no rs number assigned

The strongest and most replicated genetic risk factors for DLB are unequivocally APOE ε4 allele and Glucocerebrosidase (GBA). APOE ε4 carriers often develop mixed DLB-AD pathology. However, the ε4 allele is also over-represented in pure DLB and PDD [103]. Multiple studies have found an association of APOE ε4 with an increased risk of DLB and, recently, a greater severity of LB pathology in cases with APOE ε4 and low AD pathology has been reported [97, 104, 105]. These findings imply an involvement of APOE in the mechanism of pure LB pathology spread and not only an increased risk of developing DLB, or Aβ associated DLB. Interestingly, no association of APOE genotype is observed for PD [106].

The association of GBA and DLB is well established [107]. The GBA gene encodes a lysosomal enzyme involved in the metabolism of complex glycosphingolipids (OMIM 606463). DLB patients are 8 times more likely to be carriers of GBA mutations than controls [107]. This risk is higher than that reported for PD [108], and seems to associate with earlier age at onset, severity and disease progression. Similar to APOE, GBA is likely involved in the mechanism of LB pathology formation and/or spread, although the exact cause of this predisposition is unknown. The recently reported association of DLB with PD-linked SCARB2 emphasises the importance of lysosomal pathways in DLB [98].

DLB appears to be genetically heterogeneous, with a rare contribution of pathogenic causative mutations and relatively common risk factors, which may explain why DLB is a relatively common disorder, but with a reduced aggregation in families [97]. Our knowledge of DLB is undoubtedly evolving and interrogation of currently known risk factors will improve our understanding of DLB pathophysiology.

## Neuropathology of DLB

**Fig. 2:**
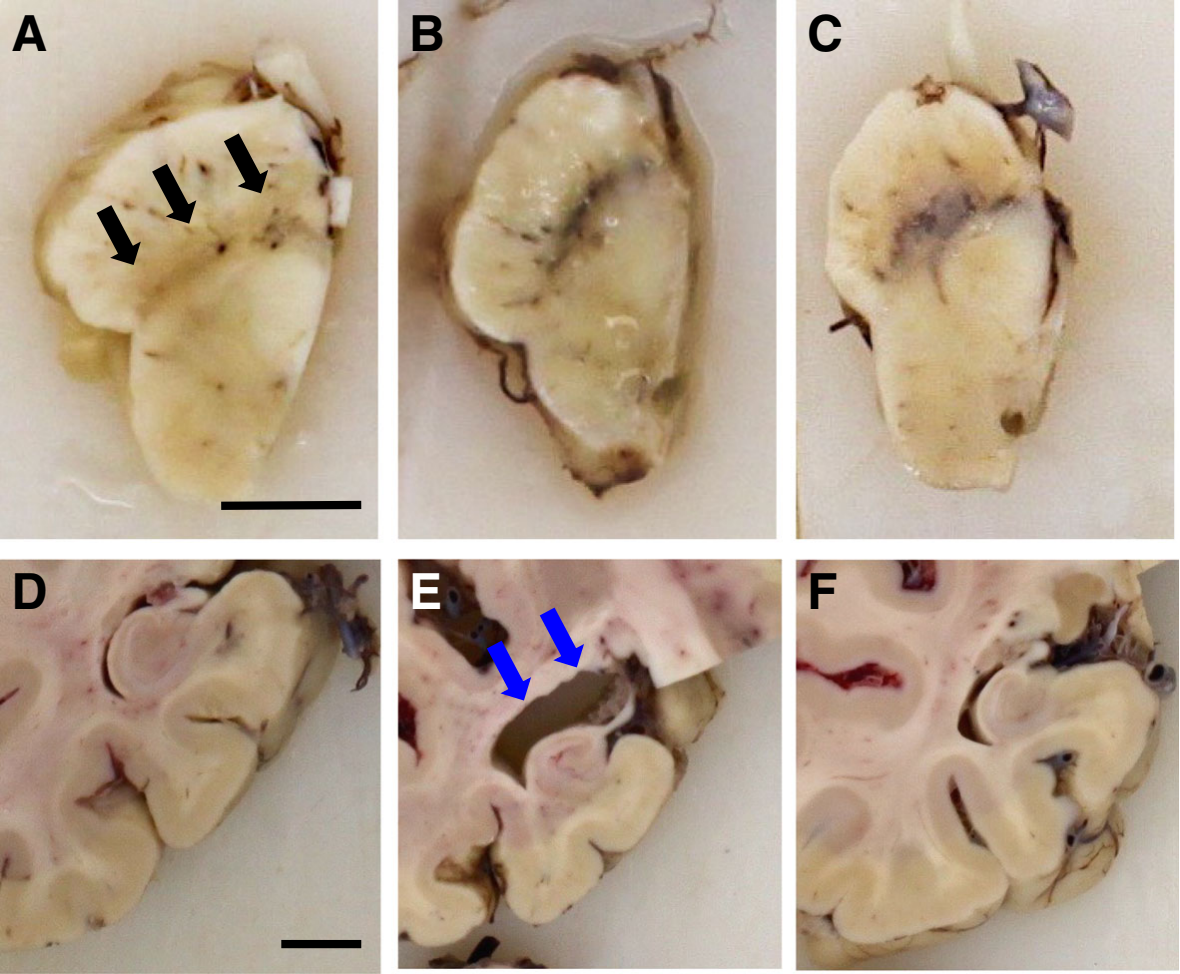
Macroscopic features of DLB. Dopaminergic cell loss is observed in the substantia nigra of a DLB patient (black arrows) (**a**) compared to AD (**b**) and control (**c**). In the same patients, atrophy of the medial temporal lobe is evident in AD, blue arrows (**e**) whilst it is relatively spared in DLB (**d**), and controls (**f**). Both scale bars represent 1cm

The majority of DLB cases show loss of pigmented, dopaminergic neurons in the substantia nigra (SN), similar to that which is seen in PD (Fig. 2a-c). However, as the main pathological changes in DLB affect the neocortex and limbic system, additional macroscopic changes are observed in patients with DLB. Some structural changes are similar to those seen in AD, with widespread cerebral atrophy being a feature of both AD and DLB [109]. Unlike AD, there is a relative preservation of the medial temporal lobe in DLB [110] (Fig. 2d-f).

**Fig. 3:**
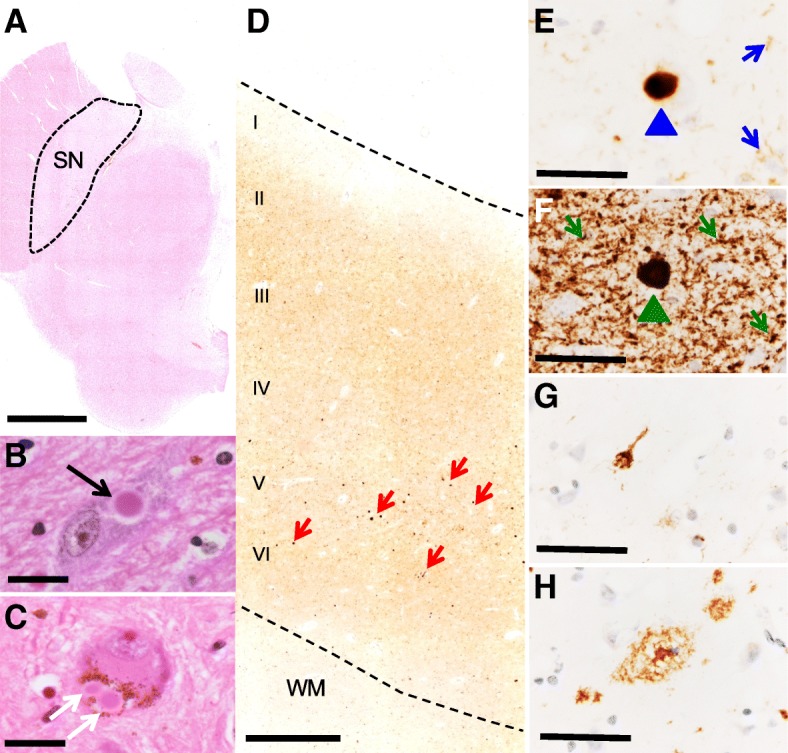
Histopathological features of DLB. Midbrain section at the level of the superior colliculus stained with H&E where dopaminergic neurons in the substantia nigra are vulnerable in DLB patients (**a**). Brainstem LBs are classically detected using H&E (**b** – black arrow) and frequently in the pigmented neurons of the SN (**c** – white arrows). Cortical LB pathology (e.g. cingulate cortex) affects all layers of the neocortex, most notably layers V and VI (**d** – red arrows). Cortical LBs and LNs can be visualised by α-syn immunohistochemistry (**e** - LB blue arrow head, LN blue arrow). α-syn phosphorylated at serine 129 detects a greater abundance of LB pathology compared to staining with phosphorylation independent antibodies (**f** - green arrows illustrate LBs, LNs, and Lewy dots). Alzheimer’s disease pathology is also a frequent finding in *post-mortem* tissue from DLB patients including hyperphosphorylated tau tangles (**g**) and Aβ plaques (**h**). Of note photomicrographs E-H were taken from sequential sections of the cingulate cortex of the same DLB patient. Abbreviations: SN, substantia nigra; WM, white matter; LB, Lewy body; LN, Lewy neurite; α-syn, α-synuclein. Scale bar represents 0.5cm in A, 20μm in B and C, 500 μm D, and 50μm in E-H

Microscopically, DLB is characterised by the abnormal accumulation of α-syn in neuronal somata and processes (*i.e*., LB and LN). Under pathological conditions, α-syn undergoes a conformational change from random coil to a cross-β sheet-rich structure [111, 112]. Electron microscopy has revealed that LB and LN are composed of unbranched α-syn filaments with a typical length of 200-600nm and a width of 5-10nm [113]. Two types of LB have been described: *i*) brainstem LB have an acidophilic and argyrophilic core with a pale stained halo, classically seen using H&E staining (Fig. 3a and b). Typically they are 8-30μm in diameter and predominantly seen in pigmented neurons of the SN (Fig. 3c); *ii*) cortical LB are eosinophilic, rounded, angular or reniform structures without a halo and can be visualized using α-syn immunohistochemistry, most notably in layers V and VI of the neocortex (Fig. 3d-f).

α-syn can undergo extensive posttranslational modifications (PTM), with phosphorylated, nitrated, and SUMOylated forms of α-syn identified in LB [114–116]. Immunohistochemistry of α-syn phosphorylated at serine 129 in DLB has revealed far more abundance of α-syn than phosphorylation-independent antibodies and, in addition to LB and LN, more threads and dot-like structures (Lewy dots) are immunopositive for this modified form of α-syn (Fig. 3f) [117, 118]. Therefore, it is tempting to speculate that cell types in individual brain regions could accumulate differently modified forms of α-syn, which may have implications in the design of disease modifying therapeutics, or in defining previously unidentified discrete clinico-pathological subtypes of DLB.

Based on current international neuropathological staging systems it is impossible to distinguish DLB from PDD, which shares similar clinical, neurochemical and morphological characteristics with DLB. However, imaging and *post-mortem* studies have suggested DLB cases exhibit elevated limbic and striatal AD related pathologies, and a lesser degree of dopaminergic cell loss compared to PDD [119–121].

The common occurrence of additional pathologies in DLB (e.g. AD related neurofibrillary tangles and Aβ plaques (Fig. 3g and h), or fronto-temporal lobar degeneration related (FTLD)) is of current interest [122–127]. The presence of multiple pathological lesions has implications for disease prognosis, and has been shown to alter the clinical phenotype; an elevated burden of hyperphosphorylated tau has been associated with a shorter survival time from the onset of dementia [128], and a summated score of hyperphosphorylated tau, Aβ, and α-syn is a better predictor of cognitive decline as measured by MMSE compared to individual pathology scores [129]. Intracellular inclusions of TDP-43 (Transactive response DNA-binding protein 43), the hallmark pathology in FTLD, are also often observed in DLB, with prevalence rates reported to be between 0-56% [127, 130, 131]. The distribution of TDP-43 pathology differs in DLB compared to FTLD, with limbic structures affected early in the degenerative process[127, 132]. The presence of TDP-43 pathology has been shown to modify the clinical and radiological findings in neurodegenerative diseases, as patients with additional TDP-43 pathology are more cognitively impaired and display greater hippocampal atrophy as seen on MRI compared to patients lacking TDP-43 pathology[133, 134]. Concomitant cerebrovascular pathology is also commonly observed, appearing in 50% of autopsy-confirmed DLB cases[125]. Reduced cerebral blood flow and microvessel density associated with decreased vascular endothelial growth factor, maybe secondary to α-syn accumulation in the occipital cortex[135], have been suggested. However, there is a still a gap in the knowledge of the exact pathogenesis of CVP in DLB and the cumulative effect on clinical phenotype. Unsurprisingly additional pathologies may impede the clinicians’ ability to provide an accurate diagnosis of DLB [24, 27, 128, 136–138].

**Fig. 4:**
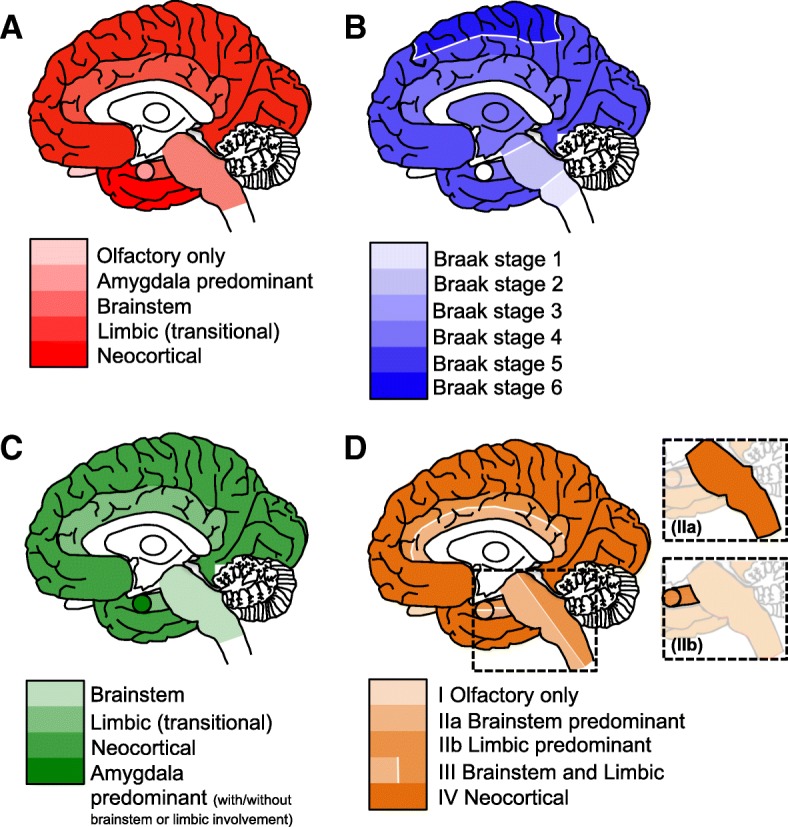
Schematic diagrams illustrating the neuropathological staging systems for LBD. The Newcastle-McKeith criteria distinguishes between brainstem predominant (regions affected including IX/X motor nucleus, locus coeruleus, and substantia nigra), limbic (transitional, regions include amygdala, transentorhinal cortex, and cingulate cortex), and diffuse neocortical (frontal, temporal, parietal, lobes are affected). N.B. the most recent consensus included the addition of olfactory only, and amygdala predominant stages [11] (**a**). Braak staging of α-syn deposition: Braak stage 1, IX/X motor nucleus of the medulla oblongata, Braak stage 2, addition of lesions to the locus coeruleus, Braak stage 3, α-syn progresses to the substantia nigra of the midbrain, Braak stage 4, α-syn lesions now detected in the transentorhinal region and CA2 of the hippocampus, Braak stage 5, higher association of the neocortex are affected, and Braak stage 6, α-syn is visible in the premotor and motor regions [139] (**b**). Leverenz and colleagues modified the original Newcastle-McKeith criteria to include cases that lack α-syn pathology in any other regions with the exception of the amygdala, known as amygdala predominant LB disease [140] (**c**). Beach and colleagues proposed a unified staging system to include cases that have α-syn confined to the olfactory bulb or bypass the brainstem to the limbic predominant pathway [41] (**d**)

There are several internationally recognised neuropathological staging systems to assess the topographical distribution of α-syn [11, 41, 139, 140] incorporating a semi-quantitative grading of α-syn to assess the severity in individual brain regions (Fig. 4). The majority of cases can be classified in accordance with the suggested rostral-caudal propagation of α-syn. However, other factors such as concomitant AD type pathology (often observed in DLB and taken into account in the fourth consensus report of the DLB Consortium [11]), or a genetic susceptibility may influence α-syn aggregation, and it is possible that certain brain regions may become more vulnerable to further abnormal protein deposition. α-syn deposits have also been detected in the peripheral nervous system of patients with synucleinopathies[141, 142]. Further investigations highlighted a multi-organ distribution of α-syn including the gastrointestinal, cardiovascular, endocrine, and respiratory systems[143]. A high prevalence of submandibular gland α-syn has been reported in autopsied-confirmed cases, with 89% / 71% of PD / DLB exhibiting α-syn positive lesions and α-syn positivity has been reported in skin nerve fibres of DLB patients [144]. However, the relation between peripheral and central nervous system α-syn pathology is not fully understood and warrants further investigation.

α-syn is assumed to spread throughout the brain in a prion-like manner [145, 146] (see section "Molecular Mechanisms"). The staging system proposed by Braak and colleagues is based on the assumption that cerebral α-syn pathology initially manifests in the medulla from where it propagates, to the SN (at which stage clinical symptoms of parkinsonism are evident), and further to the neocortex (when clinical symptoms associated with dementia emerge) [10]. However, in DLB, which initially manifests with clinical dementia and only rarely with extrapyramidal symptoms, this topographical spreading pattern is not applicable and α-syn pathology may initially manifest in limbic and/or neocortical areas. In cases with additional limbic and neocortical AD pathology, α-syn pathology may be aggravated as it is tempting to speculate that neurons already subjected to insult by concomitant tau and/ or Aβ pathology could act as trigger sites contributing to the aggregation and deposition of α-syn in the neocortex. Evidence in support of this hypothesis is provided in cases that neuropathologically fulfill criteria for DLB and AD, where the concurrent presence of hyperphosphorylated tau, Aβ, and α-syn has been demonstrated to alter the topographical distribution of pathological protein aggregates compared to cases that do not harbor multiple lesions within the same brain region [137]. The notion that hyperphosphorylated tau, Aβ, and α-syn can influence each other, promoting simultaneous aggregation, is also supported by data from in vitro and transgenic animal studies [147–151], however as this is yet to be fully recapitulated in human tissue [152], future work in this area will help to establish the presence of a mechanistic link between multiple pathologies.

The relevance of Lewy pathology to the patho-mechanisms responsible for eliciting the clinical phenotype is still controversial. Numerous clinico-pathological studies have failed to correlate LB density with disease duration, age of onset, presence or absence of cognitive fluctuations, visual hallucinations, delusions, recurrent falls, severity of parkinsonism or cognitive decline [153–156]. This is not entirely surprising, as two of the core clinical features of DLB (fluctuations in cognition and recurrent visual hallucinations) are transient in nature. Therefore, other dynamic factors (such as perhaps the levels of oligomeric species of α-syn, or specific PTMs of α-syn) may be better predictors of clinical features of DLB rather than overall LB density. Another hypothesis is that formation of LB represents a neuroprotective mechanism in affected neurons [157, 158], which may account for the lack of association in cognitive decline with increasing LB burden.

## Molecular mechanisms

Despite the controversy about the causal role of LB pathology in LBD, the aggregation of α-syn is considered a central process in all synucleinopathies. The aggregation of α-syn follows a two-step process, initiated by a rate limiting nucleation phase in which soluble monomers associate into transient intermediate oligomers, which are built upon during the exponential elongation phase, producing primary filaments that are in turn integrated into fibrillary assembles [159]. This process conforms to a generalised scheme of protein fibrillation established not only for α-syn [160] but also for other proteins such as tau [161] or Aβ [162]. The conversion between nucleation and elongation likely requires small disordered oligomeric arrangements to adopt more stable ordered configuration, resistant to degradation and capable of promoting further fibrillation [163]. Each step of fibrillation can be modulated by a number of factors including familial α-syn mutations [164–167] as well as by a variety of PTMs, such as acetylation [168], glycation [169], nitration [170], oxidation [171], phosphorylation [114, 172, 173], or truncation [174].

The initial lag phase of the primary nucleation can be bypassed by the presence of “seed competent” fibrils [175], resulting in a secondary nucleation event, which likely facilitates the formation of new aggregates on the surface of existing fibrils [176].

The apparent induction of de-novo fibrillation via the uptake of transmitted α-syn arrangements may underlie the prion-like spread of pathology initially observed as the transmission of Lewy pathology to transplanted fetal neurons [145, 177]. Additional studies demonstrated α-syn aggregates may spread between neurons by sequestering native α-syn thereby promoting aggregate growth [178].

The suggestion that α-syn may spread like a prion is an attractive hypothesis, as it may explain the stereotyped topography of Lewy pathology and clinical heterogeneity across LBD. Importantly, it has also considerable translational potential. However, the regional spread of α-syn does not appear to be solely determined by the strength of anatomical connectivity or a ‘nearest neighbor’ rule, indicating cell- or region- autonomous factors may govern the development of LB pathology [179].

The lymphocyte activation gene 3 (LAG-3) binds α-syn with high specificity and induces endocytosis from the extracellular milieu, and its knockdown impedes the cellular uptake of α-syn fibrils [180]. However, data from our group on the distribution of LAG-3 in *post-mortem* brain tissue indicate it is a pan-neuronal marker, and is expressed by neurons that do not typically manifest LB (unpublished data).

We have also recently shown that, similarly to Aβ, α-syn interacts with the prion protein (PrP), triggering a signaling cascade that culminates with neuronal dysfunction [181].

Low expression of native α-syn has been described in regions that do not develop LB pathology [182] and decreased cellular expression is prohibitive to intracellular aggregation [183]. Therefore, low expression levels of native α-syn within particular neuronal sub-types may inhibit intracellular aggregation by limiting the initiation nucleation phase.

Nevertheless, the consequences for those cells affected depends on the configuration of the prion-like agent. Somewhat surprisingly, the uptake of fibrils in vitro has been associated with a protective outcome despite accelerated aggregation, and is in contrast to the induction of apoptosis triggered upon the uptake of monomeric or oligomeric preparations [184]. Accordingly, as mentioned above, it remains unclear if mature fibrils which comprise LBs are the primary toxic agent of the disease. Indeed, whilst the presence of cortical LBs is associated with cognitive impairments [185], there is little evidence to support a correlative relationship between LB burden and the severity of dysfunction [154, 155, 186, 187]. This disconnect is not only evident symptomatically, but also at the cellular level, as key pathological changes are often reported independent and/or assumed prior to LB formation. These include synaptic dysfunction [188], decreased neurofilament mRNA production [189], the accumulation of axonal trafficked proteins [190], the induction of apoptotic cascades [191] and neuronal loss [192, 193].

Thus, despite the stable prominent nature of α-syn fibrils, it is likely that toxicity is instead driven by a pool of ill-defined heterogeneous oligomers. These oligomers may dynamically shift in equilibrium, altering their properties and substrates, either acting as intermediates of aggregation (on-pathway oligomers) or terminal assemblies (off-pathway oligomers) from which fibrillation is no longer favorable [160]. Owing to their transient nature, the investigation of oligomers has been somewhat problematic. Nevertheless a variety of oligomers have been defined by their structure, as observed in vitro. These include annular [194] and globular [184] and/or by their involvement in fibrillation [195]. A truncated breakdown product from the incomplete lysosomal processing of fibrils, so called “pα-syn*”, has recently been demonstrated as highly toxic [196], highlighting the potential for the retroactive production of toxins. Mechanistically, an array of cellular insults conducive to dysfunction and death have been attributed to α-syn oligomers; including membrane permeabilization [195, 197, 198], altered synaptic transmission and plasticity [36, 169, 181], the breakdown of protein degradation [199], as well as impairment of cellular organelles such as mitochondria and endoplasmic reticulum [196, 200–202]. Despite our progress in understanding the molecular basis of α-syn toxicity, it must be conceded that the generalised terms “oligomers” and “fibrils” lacks the fidelity required for the evaluation of physiological aggregates. Multiple conformations of these assemblies exist, which dictates their biological profile, and may account for specific strains of aggregates resulting in differential clinical diseases [203–205]. As such, the extrapolation or generalisation of outcomes observed from in vitro systems, synthetic preparations or from differing protocols of biological extractions must be made with extreme caution.

## Conclusions and outlook

DLB is a devastating disorder for which we lack effective therapies. This is, at least partly, due to our lack of detailed understanding of the molecular underpinnings of the disease. Importantly, consensus guidelines have improved the diagnosis and management of DLB, and the 1-year rule remains valid for distinguishing DLB from PDD in the clinical setting [37]. However, we still need additional guidelines (including better stratification of patient cohorts) and outcome measures for future clinical trials in DLB. In addition, we need to continue to improve our understanding of genetic factors, of neuropathological hallmarks, and of the underlying molecular mechanisms.

At the molecular level, we need to identify factors that may justify that the same proteins, such as α-syn, tau, or Aβ, may behave differently and lead to distinct disease manifestations. In this context, PTMs emerge as likely suspects, as they could influence the behavior and accumulation of the various proteins in different brain regions. Given that PTMs can be either transient or irreversible, they may operate together or independently, and may influence the formation of prion-like strains that could then spread in different ways depending on the disease.

Progress is challenging due to the considerable heterogeneity observed in DLB. The hope is that the knowledge acquired will enable us to define better biomarkers for early diagnosis and for following disease progression, and to identify novel targets for therapeutic intervention. Ultimately, our collective goal as a community, should be to distinguish DLB from other similar disorders, in order to better assist patients and families not only with disease management but also, and more importantly, modifying, stopping, or altogether prevent this terrible disease.

## Abbreviations

AD: Alzheimer’s disease
DCLB: Dementia associated with cortical Lewy bodies
DLB: Dementia with Lewy bodies
LB: Lewy body
LBD: Lewy body disease
LBVAD: LB variant of Alzheimer’s disease
PD: Parkinson’s disease
PDD: Parkinson’s disease dementia
PET: Positron emission tomography
RBD: REM sleep behavior disorder
REM: Rapid eye movement
SDLT: Senile dementia of LB type
SPECT: Single-photon emission computed tomography
α-syn: Alpha-synuclein
